# Optimal training dataset composition for SVM-based, age-independent, automated epileptic seizure detection

**DOI:** 10.1007/s11517-016-1468-y

**Published:** 2016-03-31

**Authors:** J. G. Bogaarts, E. D. Gommer, D. M. W. Hilkman, V. H. J. M. van Kranen-Mastenbroek, J. P. H. Reulen

**Affiliations:** Department of Clinical Neurophysiology, AZM Maastricht, P. Debyelaan 25, 6229 HX Maastricht, Netherlands

**Keywords:** Age independent, Electroencephalography, Epilepsy, Classification, Support vector machines

## Abstract

Automated seizure detection is a valuable asset to health professionals, which makes adequate treatment possible in order to minimize brain damage. Most research focuses on two separate aspects of automated seizure detection: EEG feature computation and classification methods. Little research has been published regarding optimal training dataset composition for patient-independent seizure detection. This paper evaluates the performance of classifiers trained on different datasets in order to determine the optimal dataset for use in classifier training for automated, age-independent, seizure detection. Three datasets are used to train a support vector machine (SVM) classifier: (1) EEG from neonatal patients, (2) EEG from adult patients and (3) EEG from both neonates and adults. To correct for baseline EEG feature differences among patients feature, normalization is essential. Usually dedicated detection systems are developed for either neonatal or adult patients. Normalization might allow for the development of a single seizure detection system for patients irrespective of their age. Two classifier versions are trained on all three datasets: one with feature normalization and one without. This gives us six different classifiers to evaluate using both the neonatal and adults test sets. As a performance measure, the area under the receiver operating characteristics curve (AUC) is used. With application of FBC, it resulted in performance values of 0.90 and 0.93 for neonatal and adult seizure detection, respectively. For neonatal seizure detection, the classifier trained on EEG from adult patients performed significantly worse compared to both the classifier trained on EEG data from neonatal patients and the classier trained on both neonatal and adult EEG data. For adult seizure detection, optimal performance was achieved by either the classifier trained on adult EEG data or the classifier trained on both neonatal and adult EEG data. Our results show that age-independent seizure detection is possible by training one classifier on EEG data from both neonatal and adult patients. Furthermore, our results indicate that for accurate age-independent seizure detection, it is important that EEG data from each age category are used for classifier training. This is particularly important for neonatal seizure detection. Our results underline the under-appreciated importance of training dataset composition with respect to accurate age-independent seizure detection.

**Electronic supplementary material:**

The online version of this article (doi:10.1007/s11517-016-1468-y) contains supplementary material, which is available to authorized users.

## Introduction

In intensive care units (ICU), many vital parameters are recorded. However, monitoring brain function by electroencephalography (EEG) is rare, mainly because signal interpretation requires expert visual inspection which is very labour intensive. One to six per cent of newborns in the neonatal ICU experiences (sub clinical) seizures. The figures for premature and low-birth-weight children are even higher [30]. It is estimated that the incidence of non-convulsive seizures (NCS) in adult patients with coma can be up to 48 % which is much higher than suggested by clinical suspicion alone [5, 8, 18, 22, 28]. Automated seizure detection is a valuable asset to health professionals, which makes early treatment possible in order to minimize brain damage. In past years, numerous neonatal seizure detection methods have been developed [1, 2, 7, 12, 17, 24]. Although reported seizure detection performance is often good enough for clinical application, there is still room for improvement. Furthermore, performance is often much worse when a classification method is actually applied in a real clinical setting [14]. Automated EEG-based seizure detection research has mainly focused on two separate aspects: EEG feature computation and classification methods [20]. As far as the authors are aware, little research has been published regarding optimal training dataset composition for epileptic seizure detection. As seizure detection performance depends on what a classifier has learned during training, the importance of an optimal training dataset is evident. To compose an optimal training dataset for epileptic seizure detection, two distinct EEG datasets were used: neonatal and adult EEG registrations. Neonatal seizure detection is considered much more difficult compared to adult seizure detection. This is because adult seizures are generally characterized by less complex waveforms, mainly due to the completed maturation of the adult brain [9]. Until now, most neonatal seizure detection procedures are based on adapted adult seizure detection systems [12]. Regarding the marked EEG differences between neonates and adults, the development of a specific neonatal classifier is warranted [4]. It has been shown that a support vector machine (SVM) classifier trained on neonatal EEG data can successfully be used for the detection of epileptic seizures in adults [9]. Moreover, our recently introduced feature baseline correction (FBC) technique compensates for differences in feature values between neonatal patients [3]. This FBC technique might also reduce differences in EEG features values between adults and neonates. If so, neonatal and adult EEG datasets can be combined to train an SVM classifier for accurate seizure detection in both adults and neonates. The objective of this paper is to evaluate the neonatal and adult seizure detection performance of three different SVM classifiers trained on: one trained on neonatal EEG, one trained on adult EEG, and one trained on both neonatal and adult EEG. This evaluation is carried out with and without FBC to investigate the relevance of FBC in age-independent seizure detection. Our overall goal is to gain insight into optimal training set composition for age-independent seizure detection. We hypothesize that FBC enables optimal age-independent seizure detection using an SVM classifier trained on the combination of neonatal and adult EEG. In addition to comparing classification performance, specific properties of the classifiers themselves will be investigated to gain more insight into the compatibility of neonatal and adult datasets. An SVM classifier consists of support vectors (SVs), i.e. those training vectors considered most relevant for classification. By analysing the SV composition of the classifiers trained on both adult and neonatal EEG, further insight can be gained into the relative importance of each patient subset for classifier training. Because of the more complex seizure waveforms seen in neonates, the neonatal dataset will likely contribute more SVs to the SVM classifier than the adult dataset.

## Materials and methods

### Dataset

The dataset used in this study consists of two different subsets. The first dataset titled *Neo* consists of 54 routine EEG registrations from 39 different neonates with a mean post-conceptional age of 39 weeks (range 28–59 weeks). The second dataset titled *Adults* consists of 41 routine registrations from 39 adult patients (mean age: 53 years, range 22–84 years). The only inclusion criterion used for both datasets was the presence of at least one epileptic seizure per recording. The EEGs were recorded between 2000 and 2014 in the Maastricht University hospital, MUMC+, in the Netherlands. Further information about the patients used in this study is available in the supplementary information. The recordings for the *Neo* dataset were made according to the international 10–20 electrode configuration system for neonates (nine electrodes) [6]. For the *Adults* dataset, the recordings were made using the full 10–20 electrode set (19 electrodes).

### Feature extraction

EEG recordings were recorded at a sample frequency of 250 Hz, band-pass filtered between 0.5 and 32 Hz and subsequently down-sampled to 25 Hz. The EEG is then partitioned into 10-s epochs with 5 s (50 %) overlap between epochs. Table 1 lists the 103 quantitative EEG features computed per epoch for each uni-polar EEG channel as described in the literature for neonatal seizure detection [13, 25]. These features are not only used for neonatal seizure detection but in many different subject groups [15]. They stem from different signal description domains such as time, frequency and information theory. Each EEG epoch is now described by a feature vector per channel. The Neo dataset is composed of 21,855 seizure and 141,277 non-seizure feature vectors (FVs). The Adults dataset is composed of 13,043 seizure and 265,470 non-seizure FVs.

**Table 1:** EEG features extracted for each epoch

EEG features
Total power (0–12 Hz)
Peak frequency of spectrum
Spectral edge frequency (SEF 80 %, SEF 90 %, SEF 95 %)
Power in 2 Hz width subbands (0–2, 1–3,…10–12 Hz)
Normalized power in same subbands
Wavelet energy (Db4 wavelet coefficient corresponding to 1–2 Hz)
Curve length
Number of maxima and minima
Root mean square amplitude
Hjorth parameters (activity, mobility and complexity)
Zero crossing rate (ZCR), ZCR of the Δ and the ΔΔ
Variance of Δ and ΔΔ
Autoregressive modelling error (AR model order 1–9)
Skewness and kurtosis
Nonlinear energy
Shannon entropy—spectral entropy, singular value decomposition entropy
Fisher information
Linear filter bank: 15 subband energies (0–2, 1–3,…14–16 Hz)
15 Cepstral coefficients
15-s order frequency filtered bank energies
Peak–peak voltage

### Feature baseline correction (FBC)

To correct for differences in EEG features, normalization is essential in patient-independent algorithms [3, 16]. EEG characteristics change with brain maturation and may differ among EEG channels [21, 27]. Furthermore, not only seizure EEG properties may differ among subjects but also their ‘baseline’ non-seizure EEG and may therefore hamper optimal SVM classifier training. Optimally, every single feature detection threshold should be the same for each patient and EEG channel. Since this is not true, FBC attempts to achieve this equality by estimating each feature’s optimal detection threshold. FBC can be seen as a form of normalization and is performed for every feature per EEG channel for each patient separately. It involves the calculation (in training phase) or estimation (in test phase) of a feature-specific (optimal) detection threshold which is then subtracted from the calculated feature value. In the training phase in case of a channel containing seizure epochs, the feature-specific threshold is calculated using the seizure annotations. For nearly every feature, a linear relation exists between the optimal threshold (Tr) and the average non-seizure feature values (aNS). This relation is quantified from the training dataset by fitting a linear regression model through the aNS-Tr points. Using this relation, Tr can be estimated when some non-seizure epochs are available to calculate aNS. A small number of visually selected (seizure and artefact free) EEG epochs from the first 3 min of the registration are used to calculate the aNS values. The number of epochs used for this baseline depends on the availability of suitable artefact and epileptiform-free EEG but are usually but not necessarily taken from the first 3 min of the registration.

### Training datasets and test procedure

This study evaluates three different training datasets: (1) neonatal EEG (*Neo*), (2) adult EEG (*Adult*) and (3) Neo and *Adult* combined (*Combi*). *Neo* consists of 4500 seizure FVs and 9000 non-seizure FVs randomly selected from 39 neonatal patients. The number of selected seizure and non-seizure FVs per patient is weighted such that each patient contributes equally to the training dataset. This is accomplished by the random selection of a maximum of 24 seizure FVs per patient per channel. This results in 2–4-min ‘seizure’ EEG depending on the number of overlapping epochs. Adult is composed similarly resulting in 8235 seizure and 9000 non-seizure FVs. The *Combi* training dataset is the combination of *Neo* and *Adult* resulting in 12,735 seizure and 18,000 non-seizure FVs. Since random sampling of training data may have an effect on classifier performance, ten Monte Carlo simulations of the complete training and testing procedure are performed. In this way, performance measure robustness and its variance are evaluated. Each Monte Carlo simulation runs with a new randomly selected set of training FVs. Final classifier performance metrics are reported as the average of all ten Monte Carlo simulations.

Training and classification are performed using ‘leave one patient out’ cross-validation (LOO) meaning that in each LOO run an SVM classifier is trained on all but one patient’s data. This classifier is then used to classify the left out patient’s data. In this way, no information from the test patient is taken into account by the training algorithm, resulting in non-biased results. It is evident that when a classifier trained on the *Neo* dataset is evaluated using the *Adult* dataset and vice versa, LOO is not applicable. Hence, only a single classifier is trained on one dataset and its performance evaluated using the other dataset.

### SVM classifier training

The classification algorithm described in this paper has been developed at the Maastricht University hospital, MUMC+ [3]. It is an improved version of the algorithm introduced to the field of neonatal seizure detection by Temko et al. [24], which is based on SVM. The improvements consist of patient-specific EEG feature baseline correction (FBC) and classifier output post-processing using a Kalman filter. A more detailed description can be found in our recent paper [3].

An SVM is a discriminative model which uses a subset of the training data to construct a surface that separates seizure from non-seizure feature vectors [23, 29]. Using a Gaussian kernel, the data are transformed from the original *N* (number of epochs) by *M* (number of features, *N* > *M*) dimensional feature space to a higher *N* by *N* dimensional space where a complex classification problem can be solved with linear discrimination functions. In this higher dimensional space, each epoch is characterized by *N* ‘new features’. Owing to the nature of the Gaussian kernel, each new feature equates to a similarity score between two epochs. Only the feature vectors that are close to the decision surface are used by the SVM. In the training dataset, each feature is normalized by subtracting the mean and dividing by the standard deviation to ensure that each feature has contributed equally to the model. The test data are normalized with the normalizing parameters from the training set. Optimal SVM classifier parameters *C* (box constraint) and *σ* (Gaussian spread parameter) are identified using a grid search for each train–test scenario. Without FBC, optimal combinations of *C* and *σ* were scattered over the grid. However, for FBC classification, the grid searches did result in a more restricted range of optimal *C* and *σ* values. Therefore, these optimal *C* and *σ* values found for FBC classification were also used for no FBC classification (Table 2). Differences in performance were relatively small and statistically not significant for a range of different *C* (10–40) to *σ* (5–8) values.

**Table 2:** Classifier performance evaluated on the *Adult*s dataset (Table 1A) and the *Neo* dataset (Table 1B), with and without FBC

A: Adults test set	No FBC	FBC	Performance increase (%)	*C*, *σ*
ACP	**0.85** **±** **0.005**	**0.93** **±** **0.002**	52	40, 7
**NCP**	**0.84** **±** **0.014**	**0.92** **±** **0.005**	52	40, 5
**CCP**	**0.83** **±** **0.04**	**0.93** **±** **0.009**	57	50, 6

B: Neonatal test set	No FBC	FBC	Performance increase (%)	*C*, *σ*
ACP	0.70 ± 0.004*^	0.86 ± 0.002*^	54	20, 8
**NCP**	**0.76** **±** 0.042*	**0.90** **±** 0.009*	59	20, 8
CCP	**0.78** **±** **0.028^**	**0.90** **±** **0.009**^	53	50, 6

Performance is expressed as average ROC AUC value and its standard deviation. The relative increase in performance due to FBC is expressed as percentage of the maximal achievable performance gain. SVM parameters *C* and *σ* were identified using a grid search for each train–test scenario. Bold values indicate which classifier results in optimal seizure detection performance. Superscript symbols indicate statistically significant differences (*P* < 0.05) between classifiers (within a column in each table) and do not apply to differences between no FBC and FBC

* Adult classifier performance (ACP) versus neonatal classifier performance (NCP)

^ ACP versus combined classifier performance (CCP)

### Classifier output post-processing

Post-processing is applied to the classifier outputs and consists of several steps. Each EEG epoch is represented by a feature vector for each channel. The SVM classifier calculates an output for each feature vector. This output represents the signed distance from the decision surface. For each epoch, the outputs are then sorted in ascending order. The sorted output time series are subsequently filtered with a Kalman filter to remove random noise. The filtered and sorted output is finally compared to a threshold to make the final classification decision.

### Performance evaluation

Seizure detection performance is evaluated in two ways: epoch-based metrics and event-based metrics. In case of epoch-based metrics, each epoch is treated as an individual observation which is classified as either seizure or non-seizure. In the testing stage, the SVM classifier is applied separately to each EEG channel. A multi-channel score is obtained by simply selecting the highest SVM classifier output of all channels. This multi-channel score is then compared to a detection threshold to obtain a binary decision: 0—non-seizure; 1—seizure. This is equivalent to requiring seizure detection in at least one EEG channel. To obtain receiver operating characteristics (ROC) curves, sensitivity is plotted versus specificity at all possible detection thresholds [10]. The area under this curve (AUC) is used to quantify the classification performance of a system and has a value of 0.5 for random classification and 1 for perfect classification. ROC curves of the ten Monte Carlo simulations are combined using vertical averaging which means that ten sensitivity values are averaged at fixed specificity values [10]. Another important factor among patients to take into account is differences in number and duration of seizures. Because AUC values are calculated for the complete dataset, it is important that final performance values are not skewed due to patients with more or longer lasting seizures. This is in particular because longer lasting seizures are usually easier to detect [26]. To prevent the results to be skewed towards patients with more and longer seizures, the dataset was balanced prior to the calculation of the AUC values. Let Nmax-S and Nmax-NS denote the number of epochs of the patient with the largest number of seizure and non-seizure epochs, respectively. A balanced dataset was achieved by randomly taking multiple copies, Nmax-S and Nmax-NS, of seizure and non-seizure epochs, respectively, per patient. As a result, each patient was weighted equally in the AUC calculation. Relative classification performance differences between FBC and no FBC are calculated as a percentage of the maximally achievable gain defined as 100 × (FBC-no FBC)/(1-no FBC). For example, an increase in AUC from 0.8 to 0.9 corresponds to 50 % relative increase.

Classification performance can also be quantified by so-called event-based metrics, i.e. ‘false detection per hour’ (FD\h) and ‘seizure detection rate’ (SDR). These metrics are clinically more relevant because the final job of an automated seizure detection algorithm is to alarm only when a seizure is detected. Whether only part of a seizure is detected is therefore of secondary importance. A false detection is defined as a single epoch or continuous segment of epochs classified as seizure without overlap with an actual annotated seizure. A seizure is detected when at least one of its epochs is classified as such.

### Statistical analysis

To apply adequate statistical testing, performance metric distributions generated by the Monte Carlo simulations are first checked for normality using the Shapiro–Wilk test as well as visual inspection [11].

In case of normal distributions, a two-sample *t* test is used; otherwise, a Wilcoxon rank sum test is used to compare classifier performance differences. *P* values below 0.05 are considered statistically significant.

### Ethical approval

All procedures performed in studies involving human participants were in accordance with the ethical standards of the institutional and/or national research committee and with the 1964 Helsinki Declaration and its later amendments or comparable ethical standards. For this type of study, formal consent is not required.

## Results

Seizure detection performance of three SVM classifiers is evaluated with and without FBC on two test datasets: neonatal EEG and adult EEG. The average AUC values with and without FBC are shown in Table 2. Several observations can be made when the different classifier and test data combinations are evaluated. First, FBC results in a relative performance increase between 52 and 59 %. Because of this distinct with and without FBC difference between classifier performance, only classification results with FBC will be presented next.

**Fig. 1:**
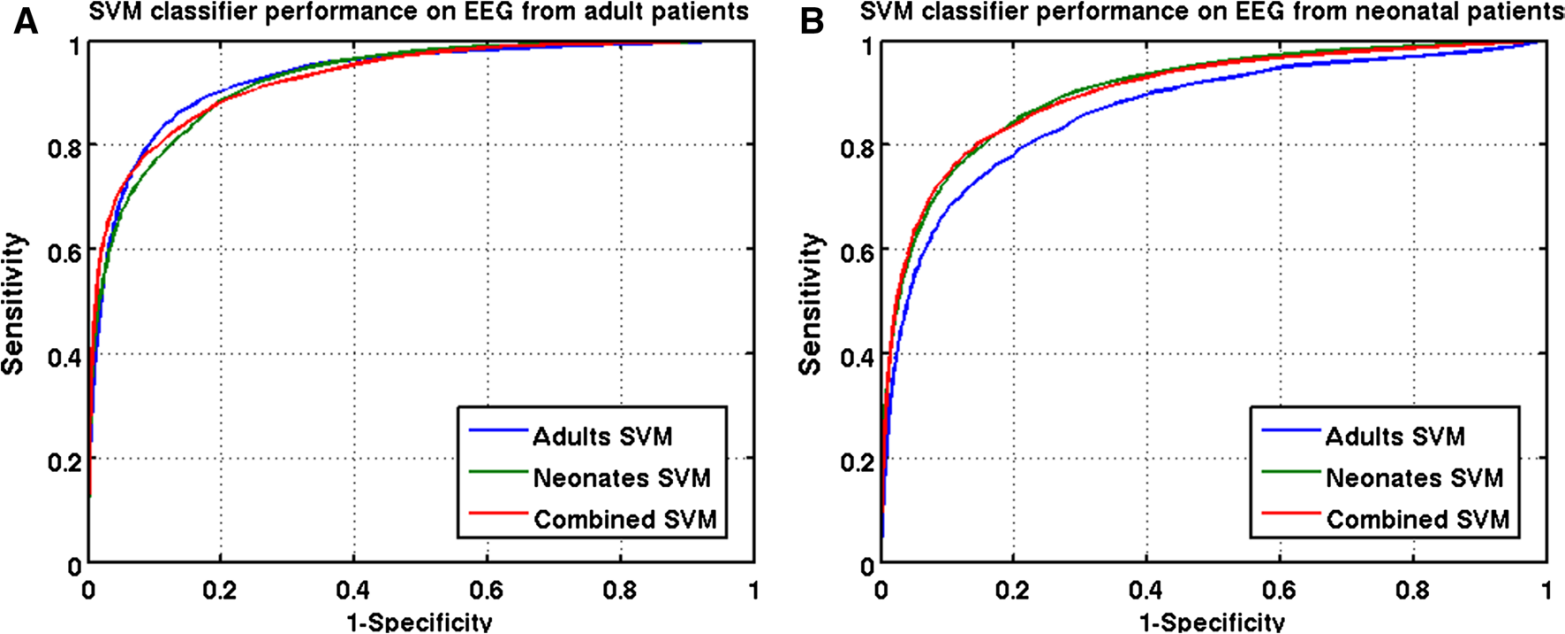
ROC curves for adult seizure detection (**a**) and neonatal seizure detection (**b**)

The average ROC curves (sensitivity versus 1−specificity) for the three classifiers are shown in Fig. 1. The ROC curves of the neonatal test set (Fig. 1b) were skewed towards higher specificity. This indicates that for neonatal seizure detection, classification of seizure epochs is more difficult compared to non-seizure epochs. This was not the case for adult seizure detection where the ROC curves were symmetric around the diagonal line (0 1, 1 0).

**Fig. 2:**
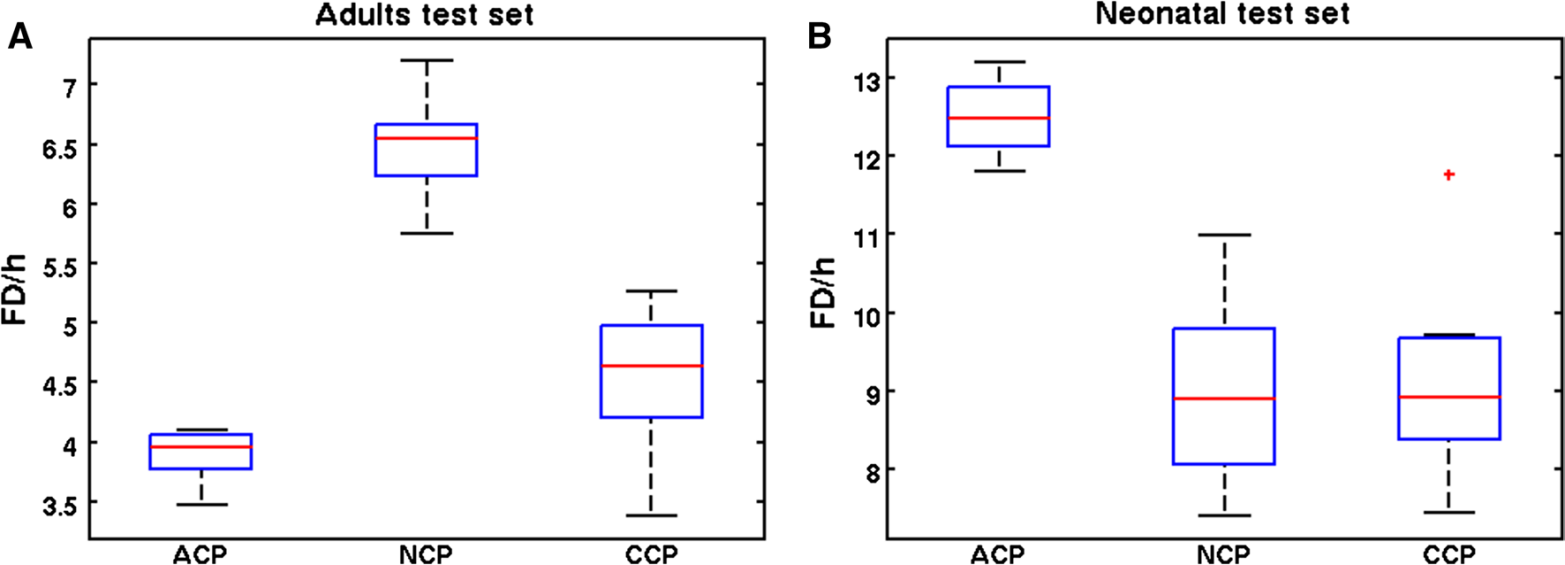
FD\h distributions belonging to a sensitivity of 80 % for adult seizure detection (**a**) and neonatal seizure detection (**b**)

In addition to the epoch-based metrics, the event-based metric FD\h is provided. The FD\h distributions corresponding to a sensitivity of 80 % are shown in Fig. 2.

The results show that neonatal seizure detection is optimal using either the neonatal SVM classifier or the combined classifier. This follows from the significantly higher AUC values (Table 2), and significantly lower number of FD\h (Fig. 2b) is found compared to the adult classifier. For adult seizure detection, the results were less unambiguous as shown in Fig. 1 and Table 2. Based on the AUC values, all three classifiers performed equally well. However, in Fig. 1a, it can be seen that the three ROC curves intersect. If high specificity is desired, the combined classifier performed best. If high sensitivity is desired, the adults classifier performed best. Furthermore, in the 60–90 % sensitivity range, NCP was lower compared to ACP and CCP. This lower NCP was also reflected in a higher FD\h rate, as shown in Fig. 2a.

In addition to classification performance, the SV composition of the combined classifier is evaluated.

SVs are those training feature vectors considered to be the most relevant for classification. It is assumed that the larger a subset’s percentage of training vectors (neonatal or adult) that become SV, the more diverse information is contained in this subset.

Without FBC, both the neonatal (49 %) and the adult (51 %) datasets account for approximately half the number of the SVs. With FBC, the amount of neonatal SVs increases to 55 %. When the SVs are evaluated per class (seizure and non-seizure) as well, the high neonatal seizure and lower adult seizure class importance becomes apparent. Of the neonatal dataset, 64 % of the seizure FVs becomes SV versus only 38 % for the adults FVs.

## Discussion

### Adult versus neonatal seizure detection

As reviewed by Ramgopal [20], scientific research mainly focuses on different EEG classification methods and features used to detect epileptic seizures. Recent research has focused on feature normalization techniques for age-independent seizure detection [3, 16]. However, little attention has been given to methods for optimal training dataset selection with respect to age-independent seizure detection [9].

This study shows that an age-independent SVM seizure detection system can successfully be used for seizure detection in both adult and neonatal patients. Despite a significant performance increase when applying FBC, neonatal seizure detection was still suboptimal using a classifier trained on adult EEG data. The same holds for adult seizure detection using a classifier trained on neonatal EEG data. However, differences in adult seizure detection performance, between the three classifiers, were much smaller compared to differences in neonatal seizure detection performance. Although AUC values for NCP (0.92), ACP (0.93) and CCP (0.93) did not differ significantly for the adult test set, FD\h was significantly lower for ACP compared to NCP and CCP. However, the metric FD\h should be approached with caution because it does not take into account the duration of each false detection [26]. Overall, in terms of both AUC values and FD\h, neonatal seizure detection performance was lower compared to adult seizure detection performance. Furthermore, we observed that when an SVM classifier was trained on both EEG data from adult and neonatal patients, more neonatal feature vectors become support vector as compared to adult feature vectors. These findings are in support of neonatal seizure detection being a more difficult task compared to adult seizure detection. This underlines the importance of including EEG data in the training data from patients with age matched to the age of the test patients, especially when used for neonatal seizure detection.

As a primary performance measure, the epoch-based area under the ROC curve was used. Monte Carlo simulation of the random training data selection provides insight into the variation in the AUC performance measures. In light of the relatively small gains in improving seizure detection, AUC variations caused by random training dataset selection can be quite significant. By using Monte Carlo simulations, variations are averaged out resulting in more robust performance measures. Without Monte Carlo simulations, one might risk drawing conclusions based on random chance. We therefore strongly recommend to quantify and to minimize variance in performance metrics when developing and evaluating seizure detection methods. Another relevant aspect is the fact that the number and/or duration of seizures among patients may differ considerable. For this reason, we corrected our results to be not skewed towards patients with more and longer duration seizures. Based on our study, we strongly recommend that with regard to seizure detection performance evaluation such a correction procedure must be applied.

### Implications and limitations

Our work shows the importance of training dataset selection for age-independent seizure detection in EEG. In a data-driven classification approach such as SVM, an EEG dataset is both used to develop and test a classification method. The role the training dataset plays in the eventual classifier performance is often neglected. As a result, it is very difficult to compare different classification methods trained on different datasets even when evaluated on the same test set. Since the availability of seizure annotated EEG registrations is often a limiting factor in the development of seizure detection algorithms, it is important to know which EEG data are the most valuable for classifier training. Our results show that FBC enables accurate age-independent seizure detection and underline the importance of neonatal seizure EEG for optimal classifier training. In this study, we have analysed a large dataset of 78 patients (adult and neonatal) with a total of 592 seizures. A limitation lies in the relatively short (~20 min) EEG registrations each containing minimally one seizure. This might not reflect a real intensive care setting where a patient is monitored for several days.

Based on the results of this study, our future research will address several matters to further improve (long term) seizure detection. The SVM–FBC classifier trained for neonatal seizure detection will be tested on a large dataset (*N* = 53) consisting of multiple day EEG registrations from adult intensive care patients as part of a neuromonitoring study. Slow changes in the EEG due to medication, level of awareness and clinical status might influence detection performance. Indeed, feature differences between patients are of similar magnitude to those within a patient over time [16]. It therefore goes without saying that using FBC with a fixed baseline is not optimal for long-term monitoring. To correct for EEG baseline variations, our future research will be focussed on the development of an automated baseline update algorithm. Moreover, it is possible to incorporate more or less online patient-specific information into the detection algorithm during long-term monitoring. When a seizure is detected, it can be added to the original training set and used to train a new, more patient-specific, classifier. Such a patient-specific classifier can be trained to detect patient-specific seizures but could also be trained to detect patient-specific false detections caused by for example periodic discharges [19]. Eventually, it might be possible to replace the non-patient-specific classifier by a patient-specific one as more information about a patient becomes available during a monitoring session. Moving from patient-independent towards patient-specific seizure detection during a monitoring session will also be part of our further research.

## Electronic supplementary material

Below is the link to the electronic supplementary material.

Supplementary material 1 (DOC 126 kb)
