# Supplementary material for: Optimal training dataset composition for SVM-based, age-independent, automated epileptic seizure detection
Source: Med Biol Eng Comput. 2016 Mar 31;54:1285–93. doi: 10.1007/s11517-016-1468-y (PMC4958398; doi:10.1007/s11517-016-1468-y)
Supplement: Supplementary file 1 — Supplementary material 1 (DOC 126 kb) [file 11517_2016_1468_MOESM1_ESM.doc]

Optimal training data set composition for SVM based, age-independent, automated epileptic seizure detection.

Supplementary information

| Patient # | Age (years) | Number of seizures | Minimal seizure duration (mm:ss) | Maximal seizure duration (mm:ss) | Registration length  (hh:mm:ss) |
| --- | --- | --- | --- | --- | --- |
| 1 | 68 | 12 | 00:40 | 02:55 | 00:21:25 |
| 2 | 26 | 3 | 02:00 | 03:20 | 00:20:05 |
| 3 | 32 | 4 | 00:15 | 02:05 | 00:19:25 |
| 4 | 60 | 17 | 00:55 | 02:50 | 00:34:25 |
| 5 | 56 | 7 | 00:25 | 00:55 | 00:47:35 |
| 6 | 47 | 1 | 02:10 | 02:10 | 00:23:55 |
| 7 | 83 | 4 | 01:10 | 02:10 | 00:43:25 |
| 8 | 75 | 13 | 01:05 | 03:00 | 00:41:25 |
| 9 | 22 | 2 | 04:20 | 08:15 | 00:26:55 |
| 10 | 57 | 4 | 00:45 | 01:15 | 00:16:25 |
| 11 | 65 | 3 | 00:50 | 01:30 | 00:26:35 |
| 12 | 65 | 5 | 01:00 | 03:10 | 00:38:55 |
| 13 | 41 | 24 | 00:45 | 10:15 | 02:15:15 |
| 14 | 46 | 1 | 00:45 | 00:45 | 00:19:15 |
| 15 | 75 | 19 | 00:15 | 01:00 | 00:29:15 |
| 16 | 40 | 12 | 00:45 | 03:30 | 00:27:15 |
| 17 | 49 | 19 | 00:40 | 03:05 | 01:33:35 |
| 18 | 50 | 2 | 02:10 | 02:30 | 00:20:15 |
| 19 | 39 | 2 | 00:45 | 02:00 | 00:23:15 |
| 20 | 66 | 2 | 02:50 | 03:05 | 00:24:45 |
| 21 | 29 | 7 | 00:10 | 01:30 | 00:46:05 |
| 22 | 65 | 3 | 01:35 | 02:15 | 00:32:35 |
| 23 | 43 | 19 | 00:10 | 00:40 | 00:19:15 |
| 24 | 37 | 2 | 00:15 | 01:45 | 00:26:45 |
| 25 | 70 | 2 | 02:35 | 02:45 | 00:43:15 |
| 26 | 84 | 16 | 00:20 | 01:00 | 00:38:25 |
| 27 | 71 | 4 | 00:30 | 02:20 | 00:21:15 |
| 28 | 88 | 7 | 00:35 | 02:05 | 00:26:25 |
| 29 | 79 | 1 | 01:30 | 01:30 | 00:21:05 |
| 30 | 29 | 3 | 00:10 | 00:10 | 00:25:55 |
| 31 | 59 | 1 | 01:10 | 01:10 | 00:31:45 |
| 32 | 83 | 6 | 00:15 | 00:25 | 00:17:55 |
| 33 | 46 | 7 | 00:20 | 02:00 | 00:26:25 |
| 34 | 23 | 5 | 00:25 | 00:35 | 00:29:55 |
| 35 | 46 | 1 | 02:00 | 02:00 | 00:42:15 |
| 36 | 50 | 2 | 00:10 | 00:10 | 00:20:05 |
| 37 | 27 | 2 | 00:11 | 00:12 | 00:20:45 |
| 38 | 68 | 2 | 02:45 | 03:05 | 00:27:25 |
| 39 | 61 | 4 | 01:05 | 01:45 | 00:19:45 |

Table A1 Patient information of the *Adult* dataset

| Patient # | Post conceptional Age (days) | Number of seizures | Minimal seizure duration (seconds) | Maximal seizure duration (seconds) | Registration length |
| --- | --- | --- | --- | --- | --- |
| 1 | 280 | 1 | 01:14 | 01:14 | 00:19:19 |
| 2 | 245 | 3 | 01:13 | 01:41 | 00:20:19 |
| 3 | 252 | 6 | 00:12 | 01:04 | 00:45:38 |
| 4 | 254 | 4 | 01:23 | 04:29 | 00:19:09 |
| 5 | 286 | 6 | 01:34 | 03:58 | 00:47:38 |
| 6 | 245 | 1 | 02:56 | 02:56 | 00:19:49 |
| 7 | 281 | 9 | 00:28 | 01:35 | 00:19:39 |
| 8 | 280 | 7 | 01:12 | 03:11 | 00:26:59 |
| 9 | 282 | 11 | 01:12 | 01:50 | 00:28:39 |
| 10 | 238 | 5 | 01:46 | 04:19 | 00:23:49 |
| 11 | 280 | 7 | 00:15 | 00:44 | 00:41:08 |
| 12 | 281 | 6 | 00:52 | 04:00 | 00:32:09 |
| 13 | 280 | 21 | 00:33 | 28:43 | 02:18:17 |
| 14 | 225 | 12 | 00:33 | 11:38 | 01:13:28 |
| 15 | 280 | 1 | 01:03 | 01:03 | 00:26:09 |
| 16 | 258 | 5 | 00:37 | 02:17 | 00:19:09 |
| 17 | 266 | 4 | 01:50 | 12:09 | 00:32:29 |
| 18 | 281 | 11 | 00:13 | 00:31 | 00:27:49 |
| 19 | 272 | 14 | 01:00 | 02:34 | 00:41:38 |
| 20 | 280 | 1 | 02:50 | 02:50 | 00:29:09 |
| 21 | 283 | 4 | 01:04 | 06:55 | 00:16:59 |
| 22 | 283 | 76 | 00:11 | 03:05 | 03:27:14 |
| 23 | 356 | 2 | 05:16 | 15:59 | 00:24:59 |
| 24 | 267 | 12 | 00:32 | 01:22 | 00:29:29 |
| 25 | 284 | 64 | 00:29 | 03:10 | 02:11:38 |
| 26 | 262 | 5 | 02:13 | 09:41 | 00:43:08 |
| 27 | 280 | 15 | 00:31 | 03:42 | 00:30:39 |
| 28 | 282 | 3 | 01:21 | 01:34 | 00:23:19 |
| 29 | ? | 3 | 00:31 | 00:54 | 00:16:39 |
| 30 | 281 | 1 | 06:10 | 06:10 | 00:25:09 |
| 31 | ? | 1 | 08:33 | 08:33 | 00:30:09 |
| 32 | 266 | 1 | 01:50 | 01:50 | 00:20:49 |
| 33 | 197 | 2 | 01:40 | 05:08 | 00:20:39 |
| 34 | ? | 1 | 00:31 | 00:31 | 00:21:39 |
| 35 | 200 | 3 | 00:39 | 01:22 | 00:19:39 |
| 36 | ? | 2 | 00:32 | 01:13 | 00:22:59 |
| 37 | 223 | 5 | 01:13 | 08:46 | 01:06:08 |
| 38 | 259 | 2 | 00:36 | 01:24 | 00:19:59 |
| 39 | 294 | 5 | 00:54 | 01:37 | 00:50:19 |

Table A2 Patient information of the *Neo* dataset
